# Adsorption Mechanisms of TM_3_ (TM = Mo, Ru, Au)-Decorated Tin Sulfide Monolayers for the Decomposition of Gas Components under Fault Conditions in Oil-Immersed Transformers

**DOI:** 10.3390/molecules29050934

**Published:** 2024-02-21

**Authors:** Min Li, Bo Wang, Hengrui Ma, Fuqi Ma, Hongxia Wang, Xiao Wang

**Affiliations:** 1 School of Computer Science and Artificial Intelligence, Wuhan Textile University, Wuhan 430200, China; 2 Hubei Engineering and Technology Research Center for AC/DC Intelligent Distribution Network, School of Electrical Engineering and Automation, Wuhan University, Wuhan 430200, China; whwdwb@whu.edu.cn (B.W.); henry3764@foxmail.com (H.M.); whumfq@whu.edu.cn (F.M.); 2018282070092@whu.edu.cn (H.W.); 3 School of Electrical and Automation, Wuhan University, Wuhan 430072, China; 4 School of Electrical Engineering, Xi’an University of Technology, Xi’an 710054, China; 5 Department of Electrical & Computer Engineering, University of Denver, Denver, CO 80208, USA; 6 School of Automation, Wuhan University of Technology, Wuhan 430072, China; xiaowang@whu.edu.cn

**Keywords:** TM_3_ (TM = Mo, Ru, Au), SnS monolayer, adsorption and gas sensing, density functional theory

## Abstract

Oil-immersed transformers play a pivotal role owing to their environmentally friendly characteristics, compact footprint, and cost-effectiveness. Ensuring the online monitoring of oil-immersed transformers is a fundamental measure to ensure the secure and stable operation of modern power systems. In this paper, metal particle cluster-doped SnS is firstly used in the adsorption and sensing of decomposition components (CO, C_2_H_2_) under fault conditions in oil-immersed transformers. The study comprehensively analyzed band structure, differential charge density, density of states, and molecular orbital theory to unveil the adsorption and sensing mechanisms of target gases. The findings suggest that the modification of metal particle clusters can enhance the surface electronic properties of single-layer SnS. In the regions of metal particle clusters and the gas–surface reaction area, electronic activity is significantly heightened, primarily attributed to the contribution of d-orbital electrons of the metal cluster structures. The modified SnS exhibits adsorption capacity in the following order: Ru_3_-SnS > Mo_3_-SnS > Au_3_-SnS. Additionally, the modified material demonstrates increased competitiveness for C_2_H_2_, with adsorption types falling under physical chemistry adsorption. Different metal elements exert diverse effects on the electronic distribution of the entire system, providing a theoretical foundation for the preparation of corresponding sensors. The findings in this work offer numerical insights for the further preparation and development of SnS nanosensors, concurrently shedding light on the online monitoring of faults in oil-immersed transformers.

## 1. Introduction

After the Second Industrial Revolution, global industries and manufacturing experienced explosive development [1,2]. Electricity, as the primary energy supply, played a crucial role in this process. Currently, the global power grid is moving towards efficiency, green solutions, and low-carbon directions, with the goal of building a globalized new energy system [3,4]. Power transformers, particularly oil-immersed transformers, have gained widespread usage due to their environmental friendliness, compact size, and high energy conversion efficiency [5,6]. In countries with abundant oil reserves, the utilization rate of these transformers is even higher. However, prolonged and uninterrupted usage of oil-immersed transformers, with their high internal oil temperature and harsh operating conditions, can lead to insulation damage and the occurrence of partial discharges [7,8]. Such internal faults in the transformer can cause significant damage to the entire power system, with far-reaching negative consequences for society and human life. Researchers have found that partial discharges in oil-immersed transformers generate a considerable amount of dissolved gases in the transformer oil, including CO, C_2_H_2_, CH_4_, H_2_, and more [9,10]. Monitoring the concentrations of these gases can provide insights into the operational status of the transformer. On the other hand, these gases also have detrimental effects on the atmosphere, posing an ongoing industrial challenge regarding their recovery and utilization. Despite studies on the adsorption and sensing of these gas molecules, issues such as low detection accuracy and poor sensing performance persist [11,12].

Since the mechanical exfoliation of graphene in 2004, research on two-dimensional materials has exploded across various disciplines such as condensed matter physics, electronic engineering, materials science, and chemistry in the past 19 years [13,14,15]. Starting with graphene, two-dimensional materials have become a diverse and extensive family, encompassing numerous members [16,17]. The unique structural characteristics and physicochemical properties of two-dimensional materials make them highly attractive for various potential applications. In the last five years, significant progress has been made in developing new synthesis methods, exploring novel structures and properties, and driving innovation and commercialization [18,19]. SnS, an IV-group metal sulfide, not only possesses abundant reserves, affordability, environmental friendliness, and good stability but also stands out as one of the rare materials exhibiting P-type semiconductor properties in its natural state [20,21,22,23,24]. Additionally, SnS has a large specific surface area, strong carrier capacity, and its bandgap and electronic properties can be controlled through nanoparticle doping [23]. These characteristics endow SnS with significant potential in optoelectronic devices, electrochemical sensors, and friction nanogenerators [25,26,27,28]. However, the development and application of SnS in existing research on two-dimensional materials have been limited, which led to the motivation for this study. In this work, we calculated the adsorption performance of pristine SnS for dissolved gases in oil and proposed SnS materials modified with transition metal (TM) trimers for online monitoring of fault gases (CO, C_2_H_2_) in transformers. Existing density functional theory studies on gas adsorption and sensing have demonstrated that introducing d-orbital electron activity through transition metal particle doping can enhance the intrinsic electron activity of the material, thereby achieving more ideal gas capture capabilities [9,29,30]. Furthermore, TM_3_ clusters are considered the most stable small cluster doping structure and have been widely adopted to enhance various material properties [31,32,33].

In recent years, transition metal modifications have been recognized as an important means of modifying material surfaces, and there are many references concerning the adsorption and evolution of small transition metal clusters. Gui et al. explored Pt cluster-modified h-BN for gas sensing and adsorption of dissolved gases in transformer oil, and they found Pt cluster-modified h-BN shows good sensitivity to C_2_H_2_ and H_2_, with decreasing conductivity in each system, but is insensitive to CH_4_ due to its weak physical sorption [30]. Moreover, Liu et al. confirmed Au_n_ (*n* = 1–3) cluster can provide a large number of adsorption active sites for MoTe_2_, improving the capture of target gases [34]. Liu et al. used Pd_4_ cluster-decorated SnO_2_ nanowire to realize the considerable detection of dissolved gases in oil. However, the adsorption and sensing behavior of dissolved gases in oil still needs to be improved [35]. Based on the current research status, this study investigated the electronic performance of TM_3_ (TM = Mo, Ru, Au)-doped SnS and analyzed the adsorption and sensing mechanisms of dissolved gases in oil on TM_3_-SnS surfaces through band structure, density of states (DOS), partial density of states (PDOS), differential charge density (DCD), and molecular orbital theory analysis. These findings lay a theoretical foundation for the application of corresponding two-dimensional material nanosensors in oil-immersed transformers. The results of this study provide numerical outcomes for the further preparation and development of SnS nanosensors while also shedding light on the online monitoring of faults in oil-immersed transformers. Furthermore, it advances the progress in the development of SnS and other related two-dimensional materials.

## 2. Results and Discussion

### 2.1. Geometric Structures and Electronic Properties of Pure SnS and TM_3_-SnS (TM = Mo, Ru, Au)

**Figure 1:**
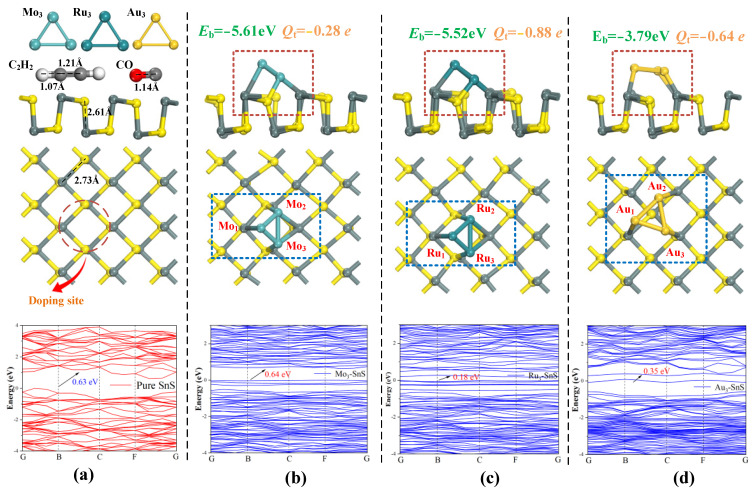
Geometric structures and electronic properties of pure SnS, TM_3_-SnS (TM = Mo, Ru, Au). (**a**) Target gases and pure SnS, (**b**) Mo_3_-SnS monolayer, (**c**) Ru_3_-SnS monolayer, (**d**) Au_3_-SnS monolayer. The *E*_b_ and band structures are also displayed in Figure 1.

Figure 1 illustrates the most stable configurations of the geometric structures employed in this work, encompassing atomic cluster models TM_3_ (TM = Mo, Ru, Au), target gas molecules, and both pristine and doped-modified monolayers of SnS. Table 1 gives the binding energy and charge transfer for different doping structures. The metal particle clusters exhibit nearly equilateral triangular shapes, highlighting their substantial stability. Both C_2_H_2_ and CO gases adopt linear structures, with initial bond lengths of 1.21 Å and 1.14 Å for C-C and C-O bonds, respectively. SnS demonstrates a two-dimensional layered configuration with alternating arrangements of Sn and S atoms, featuring a lateral stretching length of 2.73 Å and a longitudinal stretching length of 2.61 Å. Following the doping methods described in the existing literature, the hole positions on the surface of SnS were selected as the sites for doping agent placement, leading to the identification of the three most stable doping structures presented in Figure 1b,c. Under the Mo_3_, Ru_3_, and Au_3_ modification conditions, the corresponding *E*_b_ values are −5.61 eV, −5.52 eV, and −3.79 eV, indicating that the surface reaction is more intense during Mo_3_ and Ru_3_ doping, while Au_3_ doping is relatively milder. Regarding the morphological composition of the most stable doping structures, the Mo and Ru atomic clusters are inclined at a 45° angle to the doping agent plane. Furthermore, two S atoms adjacent to the doping sites sink down and form chemical bonds with the metal atoms. In contrast, when Au_3_ is modified, the three Au atoms maintain a parallel orientation with SnS, and the curvature of the substrate is less pronounced, corresponding to the magnitude of *E*_b_. The band structure of any material contributes to the understanding of its electronic properties, including the electron mobility [36]. One significant advantage of band dispersion is its capability to describe the effective mass of electrons, whether they possess finite mass or are massless. Massless electrons can only be observed in the special case of Dirac bands, resulting in exceptionally high electron mobility. From the perspective of band structure, the pristine SnS exhibits a bandgap of 0.63 eV, classifying it as a typical direct bandgap semiconductor. Following the interaction with metal particles, a significant number of impurity bands emerge throughout the doped mixture, resulting in a notable increase in the electron density of both the valence and conduction bands. The numerical calculation results indicate that the bandgaps of Mo_3_-SnS, Ru_3_-SnS, and Au_3_-SnS are 0.64 eV, 0.18 eV, and 0.35 eV, respectively. Among them, the presence of Mo_3_ metal particles has a minimal effect on the band structure, while Ru_3_ and Au_3_ doping lead to a reduction in the bandgap by 71.4% and 44.4%, respectively.

The presence of dopants introduces a large number of impurity bands and more energy levels, causing an increase in electron mobility. Specifically, the enhancement of electron mobility is primarily attributed to the contribution of d-orbital electrons from doped metal clusters, which also provides certain conditions for surface reactions with gas molecules. With the addition of metal clusters, more impurity bands appear in the band structure, which can lead to a less difficult jump of electrons; thus, nanoparticle doping enhances the surface electronic performance of SnS monolayers and augments their potential for gas adsorption.

**Figure 2:**
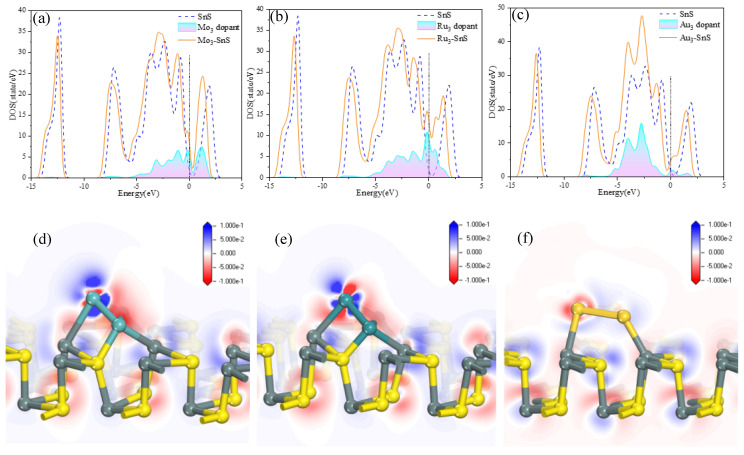
Density of states (DOS) and differential charge density (DCD) analysis of TM_3_-SnS (TM = Mo, Ru, Au). (**a**–**c**) Density of states (DOS) analysis of different doping structures; (**d**–**f**) differential charge density (DCD) analysis of different doping structures.

To comprehensively elucidate the impact of dopants on the electronic properties of monolayer SnS, Figure 2 presents the density of states (DOS) and differential charge density (DCD) before and after doping, with the dashed line representing the Fermi level. Overall, TM_3_ clusters induce a leftward shift in the DOS, particularly with distinct electron density distributions near the Fermi level, attributed to variations in the d-orbital activity of different metal particle clusters. In the Mo_3_-SnS system, the change in DOS is relatively minimal compared to the other two systems, resulting in the lowest variation in electrical conductivity. From Figure 2a, we can see that the electronic contribution of Mo_3_ is mainly distributed in the energy range from −5 eV to −2.5 eV, with multiple DOS peaks. Under the influence of the Ru_3_ cluster, there is a remarkable transformation in SnS’s DOS, and there is a substantial increase in electron filling near the Fermi level, significantly enhancing electron activity and promoting electron transitions. Additionally, the intervention mechanism of Au_3_ is similar to Mo_3_, altering the electrical conductivity of the entire system. Notably, the influence of Au_3_ on DOS extends from the −6 eV level to the Fermi level, with a peak in DOS at −2.5 eV, where electron density is noticeably higher than before gas adsorption. Figure 2b,d,f illustrate the deformation of charge density in different systems, with red regions representing electron accumulation and blue regions signifying electron depletion, using a color scale ranging from −0.1 to 0.1 e/Å. It is evident that prior to the introduction of metal atoms, the electron distribution is relatively uniform, with a slight transfer of electrons from SnS to the dopants. Clearly, an electron enrichment effect occurs at the dopant sites. The active electron layer shifts to the vicinity of the metal atom clusters before and after TM_3_ doping. Subtle geometric changes in the substrate confirm the presence of weak van der Waals forces in the chemical bonding process. Furthermore, it is visually apparent that Ru_3_ doping leads to the most significant changes in differential charge density, resulting in the maximum electron accumulation.

### 2.2. CO and C_2_H_2_ Adsorption on TM_3_-SnS (TM = Mo, Ru, Au) and Electronic Properties Analysis

**Figure 3:**
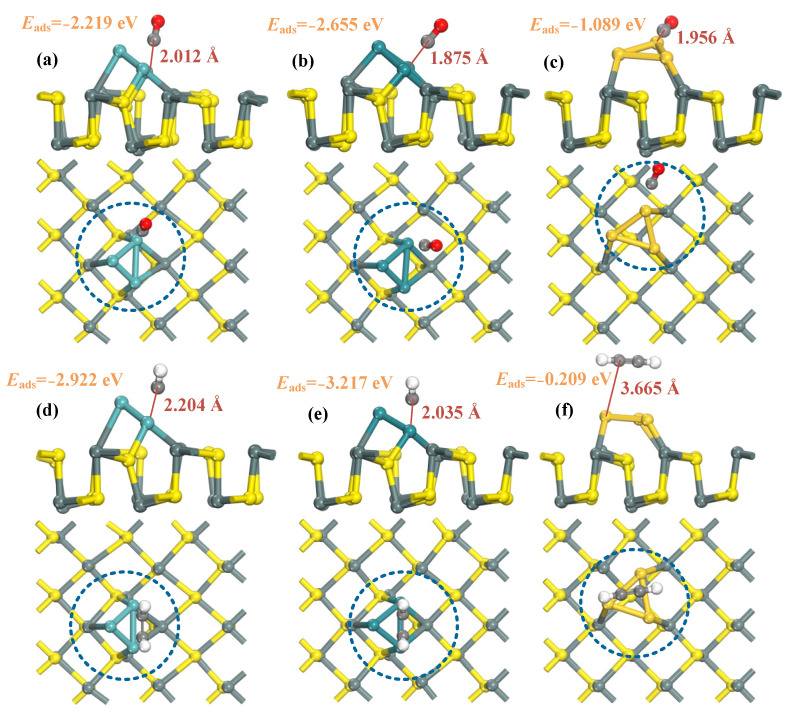
Geometric structures of CO and C_2_H_2_ adsorption on TM_3_-SnS (TM = Mo, Ru, Au). (**a**–**c**) CO adsorption; (**d**–**f**) C_2_H_2_ adsorption.

Figure 3 elucidates the most stable structures of the target gases CO and C_2_H_2_ adsorbed on the surfaces of TM3-SnS (TM = Mo, Ru, Au), accompanied by corresponding adsorption energies presented in the figure. Table 2 and Table 3 provide the adsorption energy (*E*_ads_) and adsorption distance (*d*) for each system. In the CO adsorption system, the adsorption capacities of the three mixtures are in the order of Ru_3_-SnS > Mo_3_-SnS > Au_3_-SnS, and all adsorptions fall within the realm of physical-chemical adsorption. In addition, the higher the number of electrons in the outermost d-orbitals of the dopant, the more drastic the adsorption reaction is. The adsorption energies for the three systems are −2.655 eV, −2.219 eV, and −1.089 eV, with corresponding adsorption distances of 1.857 Å, 2.012 Å, and 1.956 Å, respectively. These findings suggest that Ru_3_-SnS boasts an adsorption capacity of over twice that of Au_3_-SnS, primarily attributed to the heightened d-orbital electron activity of Ru atoms. Examining the geometric structure of the adsorption model, CO in the CO/Mo_3_-SnS system adopts a perpendicular morphology captured by Mo atoms, whereas in the CO/Ru_3_-SnS and CO/Au_3_-SnS systems, the angle between CO molecules and the horizontal substrate gradually diminishes. Furthermore, in all three adsorption systems, CO molecules approach the substrate with the C atom, as the outer electron distribution of the C atom facilitates the formation of reliable chemical bonds with transition metal atoms. Among the three adsorption systems, only in CO/Ru_3_-SnS did the C and Ru atoms form a C-Ru bond, while in the other two systems, none of the metal atoms bonded to the CO molecule. In addition, CO is activated during the surface reaction, accompanied by the C-O bond length being pulled up 0.098 Å, 0.107 Å, and 0.065 Å in the Mo_3_-SnS, Ru_3_-SnS, and Au_3_-SnS adsorption systems.

For the C_2_H_2_ adsorption system, the adsorption capacities of the three mixtures follow this hierarchy: Ru_3_-SnS > Mo_3_-SnS > Au_3_-SnS. Ru_3_-SnS and Mo_3_-SnS exhibit chemical adsorption with C_2_H_2_, while Au_3_-SnS only displays weak physical adsorption. The adsorption energies for the three systems are −3.217 eV, −2.922 eV, and −0.209 eV, with corresponding adsorption distances of 2.035 Å, 2.204 Å, and 3.665 Å, respectively. In the C_2_H_2_/Ru_3_-SnS and C_2_H_2_/Mo_3_-SnS systems, C_2_H_2_ molecules undergo significant activation, and the two H atoms are no longer aligned with the C atom. In contrast, Au_3_ doping has a minimal impact on the adsorption performance of SnS for C_2_H_2_, with an adsorption distance as high as 3.665 Å; therefore, Au_3_-SnS is not suitable for C_2_H_2_ adsorption and sensing. Overall, TM_3_-SnS (TM = Mo, Ru, Au) exhibits excellent adsorption performance for fault gases in transformers, particularly in the selective adsorption of C_2_H_2_. Different metal dopants result in variations in adsorption capacity, with Ru_3_ showing the most significant improvement in adsorption capacity, while Au_3_ has a relatively minor impact on the substrate material. In addition, none of the C_2_H_2_ formed a significant chemical bond with the mixture in three adsorption systems; however, the reaction strengths are all higher than those of the CO molecule. C_2_H_2_ is also activated during the surface reaction, accompanied by the C-H bond length being pulled up 0.078 Å, 0.099 Å, and 0.014 Å in the Mo_3_-SnS, Ru_3_-SnS, and Au_3_-SnS adsorption systems. Finally, we selected three typical conventional metal-modified materials to compare the adsorption capacity of C_2_H_2_, and the results indicate that the TM_3_ (TM = Mo, Ru)-decorated tin sulfide monolayer possesses stronger adsorption competitiveness.

**Figure 4:**
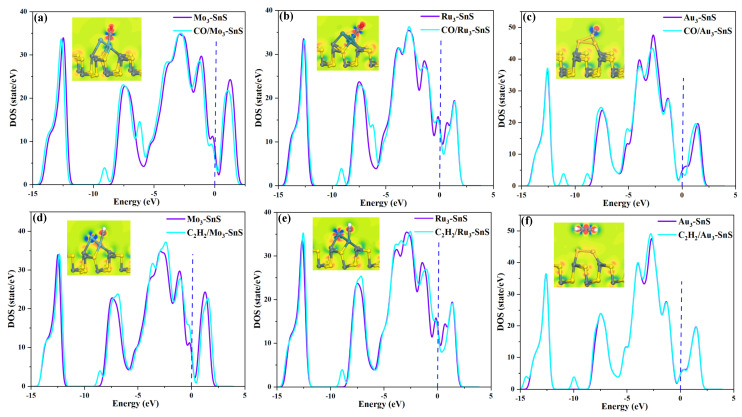
Density of states (DOS) of different adsorption systems. (**a**–**c**) DOS of CO systems, (**d**–**f**) DOS of C_2_H_2_ systems.

Density of states (DOS) is a critical tool for analyzing electronic properties, especially in revealing the distribution patterns of electronic states at different energy levels, ultimately elucidating the nature of gas adsorption. To further elucidate the adsorption mechanism of target gases on TM_3_-SnS (TM = Mo, Ru, Au), Figure 4a–f illustrate the density of states (DOS) distributions following the adsorption of CO and C_2_H_2_, and the blue dashed line in the figure indicates the Fermi level. DOS reflects the electron density at each energy level, and the higher its peak, the higher the electron density, which can lead to a rapid flow of electrons. It is evident that CO adsorption results in a decrease in electron density near the Fermi level, leading to a reduced filling of electrons between the conduction and valence bands. Such DOS change will make the flow of electrons more difficult, thereby reducing the conductivity of the entire system. Additionally, the three dopants exhibit slightly varied effects on electron distribution: Mo_3_ primarily reduces electron density to the left of the Fermi level, whereas Ru_3_ and Au_3_ dopants mainly impact electron density to the right of the Fermi level. Furthermore, the introduction of CO induces a DOS peak in the range of −10 eV to −7.5 eV for Mo_3_-SnS and Ru_3_-SnS, primarily attributed to the influence of d orbitals of metal cluster particles.

Concurrently, the reactivity of electrons in the region of the adsorption reaction significantly increases, aligning with electron transfer during the adsorption process. Compared to the CO adsorption system, the overall change in DOS during C_2_H_2_ adsorption is more subtle. Particularly, as C_2_H_2_ approaches the Au_3_-SnS surface, the DOS before and after gas adsorption almost overlap, indicating a weaker reactivity between C_2_H_2_ and the substrate. Conversely, when C_2_H_2_ approaches Mo_3_-SnS, an increase in DOS on the left side of the Fermi level is observed, facilitating electron transition from the top of the valence band to the bottom of the conduction band, resulting in an overall increase in the system’s conductivity.

In the C_2_H_2_/Ru_3_-SnS system, the addition of C_2_H_2_ leads to a slight decrease in the DOS to the right of the Fermi level, unfavorably impacting electron flow and transfer. Density functional theory (DFT) calculations suggest that C_2_H_2_ acts as an electron acceptor during surface binding, contributing to a significant increase in the electron-active layer near C_2_H_2_. Metal cluster particle-modified SnS markedly enhances the capture ability for CO and C_2_H_2_ while activating C_2_H_2_ molecules. The influence of different metal elements on electron activity varies, with Ru atoms exerting the strongest effect, Au atoms the weakest, and Mo atoms falling between the two.

During the adsorption of CO and C_2_H_2_, a greater number of impurity states appear at multiple energy levels, leading to changes in electron filling at different energy levels and resulting in distinct electronic signals. This phenomenon constitutes the fundamental basis for gas detection.

**Figure 5:**
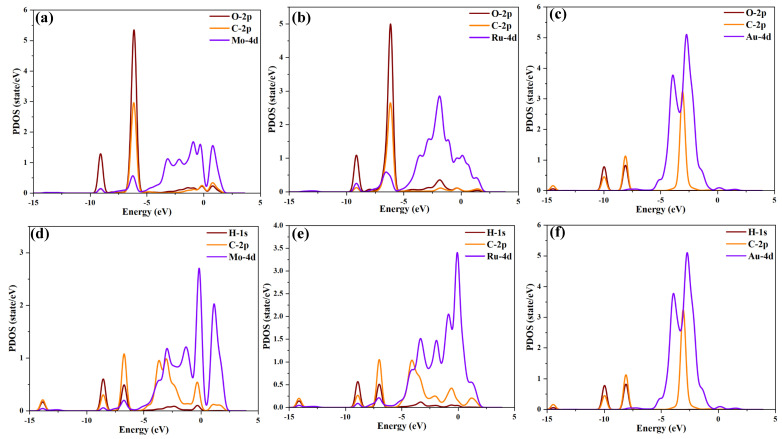
Projected density of states (PDOS) of different adsorption systems. (**a**–**c**) PDOS of CO systems, (**d**–**f**) PDOS of C_2_H_2_ systems.

The PDOS can further reflect the fundamental reasons for the changes in DOS, revealing the systematic variations in electronic properties before and after gas adsorption. Therefore, the PDOS distribution after the adsorption of target gases on TM_3_-SnS (TM = Mo, Ru, Au) surfaces is shown in Figure 5a–f. When CO is adsorbed on Mo_3_-SnS and Ru_3_-SnS, PDOS peaks simultaneously appear at the −6 eV energy level for the O-2p orbit, C-2p orbit, and Mo Ru-4d orbit. This indicates a strong orbital hybridization between the adsorbed CO and TM_3_-SnS, confirming that CO adsorption is a form of physicochemical adsorption. The distribution of the 4d orbits of metal atoms shows significant differences, with the concentration order of 4d orbits being Au > Ru > Mo, confirming the trend of DOS changes. Specifically, the Mo-4d orbital distribution ranges from −10 eV to −2.5 eV, and the Ru 4d orbital distribution is also concentrated in the −10 eV to −2.5 eV range, but with a higher concentration than Mo atoms. In the C_2_H_2_ adsorption system, peaks appear at multiple energy levels for the H-1s orbit, C-2p orbit, and TM-4d orbit, indicating strong orbital hybridization and significant adsorption reactivity between C_2_H_2_ and TM_3_-SnS. Unlike CO adsorption, the 4d orbitals of TM_3_ in C_2_H_2_ adsorption show greater dispersion, implying that gas adsorption has a greater impact on electronic distribution. In summary, the electronic activity of the metal particle’s 4d orbitals is the fundamental reason for enhancing the adsorption capacity of SnS, and different metal elements have a differentiated impact on the overall electronic distribution of the system.

### 2.3. Molecular Orbital Theory Analysis of TM_3_-SnS (TM = Mo, Ru, Au)

**Figure 6:**
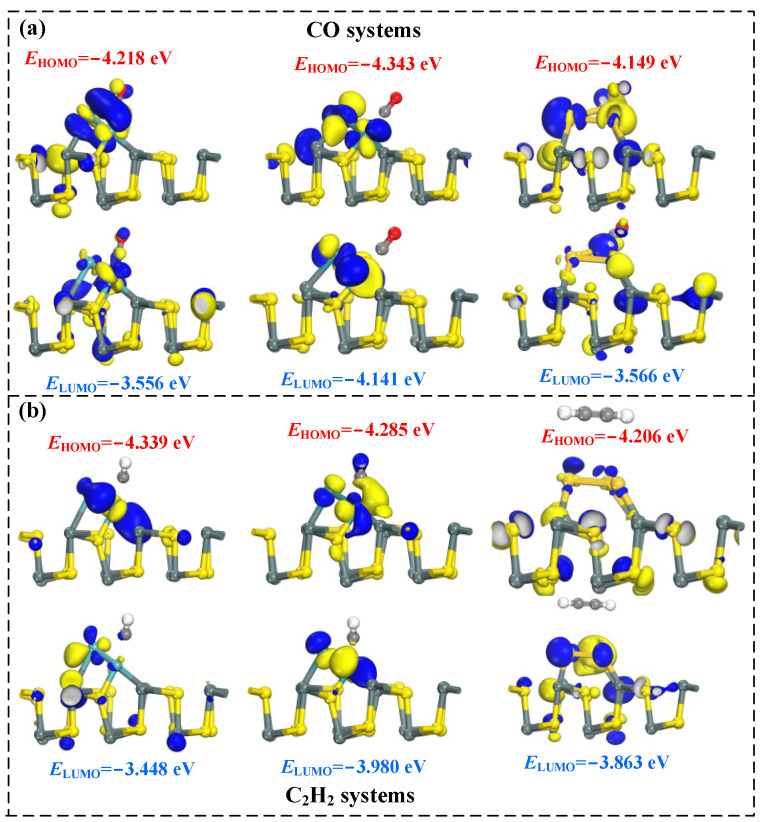
The HOMO and LUMO distribution of different adsorption systems. (**a**) The HOMO and LUMO distribution of CO systems, (**b**) The HOMO and LUMO distribution of C_2_H_2_ systems.

**Figure 7:**
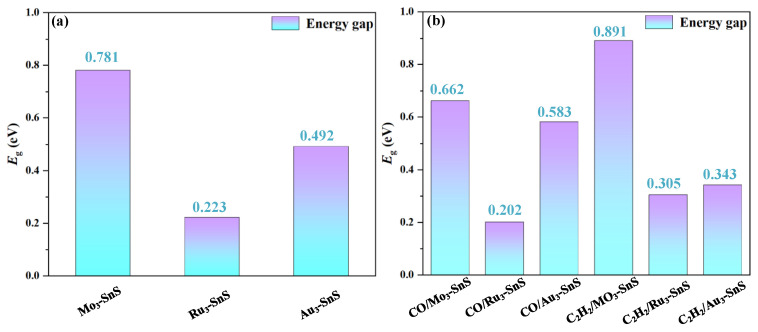
The energy gap value of different adsorption systems. (**a**) Energy gap of doped SnS, (**b**) Energy gap of adsorption systems.

In this section, we conducted an analysis and discussion of the molecular orbital theory before and after gas adsorption, with HOMO and LUMO presented in Figure 6 and the energy gap values in Figure 7. In the three doped systems (TM_3_-SnS), the HOMO values after CO adsorption are −4.218 eV, −4.343 eV, and −4.149 eV, respectively, while the corresponding LUMO values are −3.556 eV, −4.141 eV, and −3.566 eV. It is evident that a significant amount of HOMO and LUMO is distributed around the dopant and adsorbed gas molecules, indicating pronounced electronic activity in the surface reaction region and confirming the physical-chemical nature of CO adsorption. On the other hand, after C_2_H_2_ adsorption, the HOMO values are −4.339 eV, −4.285 eV, and −4.206 eV, while the corresponding LUMO values are −3.448 eV, −3.980 eV, and −3.863 eV. Clearly, only in the C_2_H_2_/Ru_3_-SnS system are HOMO and LUMO present around the adsorbed C_2_H_2_, suggesting that this system undergoes the most intense adsorption reaction, with the largest charge transfer during the process.

Furthermore, according to the computational results, the absolute value of the rate of change of the *E*_g_ values for TM_3_-SnS (TM = Mo, Ru, Au) after CO adsorption is 15.24%, 9.41%, and 18.50%, respectively. In the cases of Mo_3_ and Ru_3_ modification, the overall conductivity of the system increases after CO adsorption, while in the case of Ru_3_ modification, the overall conductivity of the system decreases. After C_2_H_2_ adsorption, the absolute value of the rate of change of the *E*_g_ values for TM_3_-SnS (TM = Mo, Ru, Au) is 14.08%, 36.77%, and 30.28%, respectively, indicating that the direction of conductivity change is opposite to that during CO adsorption. This is speculated to be mainly related to the type of gas adsorption, and the degree of activation of C_2_H_2_ during the adsorption process is also a major factor contributing to this phenomenon. Molecular orbital theory analysis reveals significant differences in molecular orbitals after the adsorption of different target molecules, indicating the strong selectivity and adsorption capability of TM_3_-SnS (TM = Mo, Ru, Au) for CO and C_2_H_2_. This makes it highly suitable for the preparation of CO and C_2_H_2_ gas adsorbents or sensors for applications in oil-immersed transformers.

## 3. Computational Details

All calculations in this paper were conducted using the Dmol^3^ module within the Materials Studio 2019 software, employing the first-principles method [37,38]. Generalized gradient approximation (GGA) with the Perdew Burke Ernzerhof (PBE) function was taken to handle the electronic energy of exchange correlation [34,39,40], and the used functional is PBE-D2. It is found that faster convergence and lower errors in the computational results can be achieved under 7 × 7 × 1 and 10 × 10 × 1 k point conditions; thus, geometric optimizations and diverse energy calculations were performed with Monkhorst–Pack special k-point meshes of 7 × 7 × 1 and 10 × 10 × 1 [41]. Atomic orbitals were represented using the double numerical plus polarization (DNP) basis set, while the valence and core electrons were described employing DFT semi-core pseudopotential (DSSP). To account for long-range interaction forces between dopants and the substrate, dispersion corrections were incorporated using the Grimme method [10,29]. For the investigation of gas adsorption and sensing mechanisms, a supercell of SnS, containing 18 Sn atoms and 18 S atoms, was constructed. A vacuum layer of dimensions 13 Å × 13 Å × 18 Å was included to prevent interference from neighboring cells [42]. Convergence criteria for energy, maximum force, and displacement were set at 10^−6^ Ha, 2 × 10^−3^ Ha/Å, and 5 × 10^−3^ Å, respectively [43,44]. Static electronic structures were calculated using a self-consistent cycle energy cutoff of 10^−6^ Ha, along with a global orbital cutoff radius of 5.0 Å [45]. During any surface reaction, the more energy released by the system means that the structure acquired after the spontaneous reaction is more stable. The determination of the aforementioned energy is mainly based on Equations (1) and (2). We built a large number of original models, and the most stable structure was obtained through optimization and energy comparison, which was put in this work.

Surface modification is accompanied by energy changes; during this process, the binding energy (*E*_b_) for TM_3_-SnS was defined as the following:

*E*_b_ = *E*_TM3-SnS_ − *E*_TM3_ − *E*_SnS_ (1)

where *E*_TM-SnS_, *E*_TM3_, and *E*_SnS_ represent the total energy of TM_3_-SnS, isolated TM_3_ (TM = Mo, Ru, Au), and pure SnS, respectively.

In addition, the adsorption energy (*E*_ads_) of the gas adsorption structure was calculated by Equation (2). A negative value of *E*_ads_ indicates that the reaction can proceed spontaneously.

*E*_ads_ = *E*_Gas/TM3-SnS_ − *E*_TM3-SnS_ − *E*_Gas_ (2)

where *E*_Gas/TM3-SnS_, *E*_TM3-SnS_, and *E*_Gas_ represent the total energy of the adsorbed structures, the modified system (TM_3_-SnS), and the individual gas molecule, respectively.

The charge amount can be achieved through Millikan charge analysis, and the charge transfer (*Q_t_*) was defined by the following:

*Q_t_ = Q*_a_ − *Q*_b_ (3)

where *Q*_a_ and *Q*_b_ represent the total charge of gases after and before adsorption, respectively.

Molecular orbital theory is also applied to this paper. The energy gap *E*_g_ of the molecular orbit is calculated by Equation (4).

*E*_g_ = |*E*_LUMO_ − *E*_HOMO_| (4)

where *E*_LUMO_ represents the energy of the lowest unoccupied molecular orbital (LUMO), and *E*_HOMO_ shows the energy of the highest occupied molecular orbital (HOMO).

## 4. Conclusions

In this work, the adsorption mechanisms of a TM_3_ (TM = Mo, Ru, Au)-decorated tin sulfide monolayer for decomposition of gas components under fault conditions in oil-immersed transformers is deeply explored based on the density functional theory. Band structure, differential charge density, density of states, and molecular orbital theory were analyzed. The following points are the primary conclusions:

(1) The presence of Mo_3_ metal particles has a minimal effect on the band structure, while Ru_3_ and Au_3_ doping lead to a reduction in the bandgap by 71.4% and 44.4%, respectively. Nanoparticle doping enhances the surface electronic performance of the SnS monolayer and augments its potential for gas adsorption.
(2) The modified SnS exhibits adsorption capacity in the order of Ru_3_-SnS > Mo_3_-SnS > Au_3_-SnS, the adsorption competitiveness of the mixture for C_2_H_2_ is better than that of CO, and C_2_H_2_ adsorption is physico-chemical while CO is physically adsorbed.
(3) The electronic activity of the metal particle’s 4d orbitals is the fundamental reason for enhancing the adsorption capacity of SnS, and different metal elements have a differentiated impact on the overall electronic distribution of the system.
(4) Molecular orbital theory analysis reveals significant differences in molecular orbitals after the adsorption of different target molecules, indicating the strong selectivity and adsorption capability of TM_3_-SnS (TM = Mo, Ru, Au) for CO and C_2_H_2_.

The findings in this work offer numerical insights for the further preparation and development of SnS nanosensors, concurrently shedding light on the online monitoring of faults in oil-immersed transformers.

## Figures and Tables

**Table 1:** Binding energy (*E*_b_) and charge transfer (*Q_t_*) of TM_3_-SnS (TM = Mo, Ru, Au).

Structure	*E*_b_ (eV)	*Q_t_* (*e*)
Mo_3_-SnS	−5.61	−0.28
Ru_3_-SnS	−5.52	−0.88
Au_3_-SnS	−3.79	−0.64

**Table 2:** Adsorption energy (*E*_ads_) and adsorption distance (*d*) of TM_3_-SnS (TM = Mo, Ru, Au).

Structure	*E*_ads_ (eV)	*d* (Å)
CO/Mo_3_-SnS	−2.219	2.012
CO/Ru_3_-SnS	−2.655	1.875
CO/Au_3_-SnS	−1.089	1.956
C_2_H_2_/Mo_3_-SnS	−2.922	2.204
C_2_H_2_/Ru_3_-SnS	−3.217	2.035
C_2_H_2_/Au_3_-SnS	−0.209	3.665

**Table 3:** Comparison of the present work with the other literature on C_2_H_2_ adsorption energy (*E*_ads_).

Structure	*E*_ads_ (eV)	*d* (Å)
C_2_H_2_/Mo_3_-SnS	−2.922	2.204
C_2_H_2_/Ru_3_-SnS	−3.217	2.035
C_2_H_2_/Au_3_-SnS	−0.209	3.665
C_2_H_2_/Pd_4_-SnO_2_ [35]	−1.282	2.108
C_2_H_2_/Pd-MoS_2_ [9]	−1.118	2.151
C_2_H_2_/Pt_3_ cluster h-BN [30]	−2.160	1.970

## Data Availability

Data are contained within the article.
